# Identification of Relatively Weak Areas of Planar Structures Based on Modal Strain Energy Decomposition Method

**DOI:** 10.3390/ma15186391

**Published:** 2022-09-14

**Authors:** Dongwei Wang, Kaixuan Liang, Panxu Sun

**Affiliations:** School of Civil Engineering, Zhengzhou University, Zhengzhou 450001, China

**Keywords:** planar structures, vibration mode, modal strain energy, relatively weak areas, quantification and visualization

## Abstract

Identifying relatively weak areas is of great significance for improving the seismic reliability of structures. In this paper, a modal strain energy decomposition method is proposed, which can realize the decoupling of the comprehensive modal strain energy of a planar structure into three basic modal strain energies. According to the decomposition results, the modal strain energy decomposition diagram and the modal strain energy cloud diagram can be drawn so as to realize the quantitative and visual analysis of the vibration modes. The method is independent of load cases and can identify relatively weak areas of a structure from the perspective of inherent characteristics. The comparison with the shaking table test results of the two-story shear wall shows that the modal strain energy decomposition method can effectively identify the type of the relatively weak area of a structure and locate the position of the relatively weak area. Finally, the 6-story shear wall is analyzed by the modal strain energy decomposition method, and the relatively weak areas under the first two vibration modes are identified.

## 1. Introduction

The weak areas of a structure may be the to be damaged in an earthquake. Several examples show that, in earthquakes [1,2,3], the damage to a structure is mainly caused by the domino effect, which starts with localized damage in weak areas and eventually leads to overall damage or collapse. The method of effectively identifying weak areas and taking targeted strengthening measures is of great significance to the analysis and design of structures.

Generally speaking, the identification of structurally weak areas needs to be carried out under certain load conditions. For example: (1) based on the accumulation of a large number of engineering cases (including earthquake damage examples), important identification experience is formed [4]; (2) the structural details are checked by mechanical tests [5]; (3) through theoretical analysis, the parameter indices of key parts are rechecked (under specific load conditions) to identify weak areas [6]. However, different load cases will result in different identification results, and it is often difficult to perform a complete analysis of load cases.

According to the d’Alembert equation, the frequency and mode shape are inherent, characteristic parameters of a structure, related only to mass and stiffness, not to load cases. Some scholars have thus used frequency to identify the weak areas of a structure [7,8]. At present, modal eigenvalues have been widely used in structural damage identification [9,10], health monitoring [11,12], and dynamic analysis [13,14]. However, the frequency of a structure represents the distribution characteristics of the absolute stiffness and absolute mass of the structure. Structural analysis using eigenvalues usually only yields quantitative thresholds (such as the critical load, the strength of key interfaces of a structure, etc.), and it is not very effective for identifying relatively weak areas of the structure.

The vibration mode vector is made up of the distribution characteristics of the relative stiffness and relative mass of a structure, and it is an ideal index for analyzing the relative stiffness of a structure [15]. Guan et al. [16] analyzed the dynamic response results of the temporary middle wall of a tunnel and found that this area was easily damaged under impact loading. Using modal analysis, Mazanoglu and Kandemir-Mazanoglu [17] found that a frame structure with column cracks had local vibration modes, and that this area was vulnerable to damage by earthquakes. At present, the vibration mode participation mass coefficient method [18] and the animation observation method are mainly used to roughly identify vibration modes. However, it is difficult to use traditional methods to quantitatively analyze vibration modes. Thus, it is difficult to achieve the purpose of effectively identifying the relatively weak areas of a structure.

Hence, a modal strain energy decomposition method is proposed in this paper. The proposed method does not involve load cases, and it can analyze a structure from the perspective of inherent characteristics. It can be applied to the vibration mode analysis of a structure and decompose the comprehensive modal strain energy so as to realize the quantitative visualization analysis of the vibration mode and the identification of the weak areas. The body of this paper is divided into five main sections. Section 2 presents the modal strain energy decomposition method of a planar square element. Section 3 uses the modal strain energy decomposition method to draw the modal strain energy decomposition diagram and the modal strain energy cloud diagram of a two-story shear wall with openings, and then it compares that with the crack diagram of the shear wall in a shaking table test. Section 4 takes a 6-story shear wall with openings as an example to decompose the modal strain energy of the first two vibration modes. Section 5 concludes the presentation of the research on which this paper is based and proposes topics for future research.

## 2. Modal Strain Energy Decomposition of a Planar Square Element

The free vibration equation of an undamped planar structure with *N* nodes is

(1) $$M\cdot \frac{d^{2}u\left(t\right)}{dt^{2}}+Κ\cdot u\left(t\right)=0$$

where **M** and **K** are the mass matrix and the stiffness matrix of the structure, respectively; $u\left(t\right)=[x_{1}\left(t\right),\;\;y_{1}\left(t\right),\;\;x_{2}\left(t\right),\;\;y_{2}\left(t\right),\;\;\ldots ,\;\;x_{N}\left(t\right),\;\;y_{N}\left(t\right)]$ is the nodal displacement vector, which contains 2*N* components, that is, the displacements of *N* nodes in the X and Y directions; $\frac{d^{2}u\left(t\right)}{dt^{2}}$ is the nodal acceleration vector; and ***0*** is a 2*N*-dimensional zero vector.

The corresponding eigenvector is expressed as

(2) $$\Phi =\left[\begin{array}{cccc} \phi_{1} & \phi_{2} & \ldots & \phi_{N} \end{array}\right]\;\left(j=1,\;2,\;\ldots ,\;N\right)$$

where $\phi_{j}$ is the *j*-th eigenvector.

Element *k* is extracted from the structure. The modal strain and modal stress of the *j*-th eigenvector satisfy

(3) $$\varphi_{k,j}^{T}B_{k}{}^{T}D_{k}B_{k}\varphi_{k,j}=\sigma_{x}\varepsilon_{x}+\sigma_{y}\varepsilon_{y}+\tau_{xy}\gamma_{xy}\;\left(j=1,\;2,\;\ldots ,\;N\right)$$

where $\varphi_{k,j}$ is the displacement vector of the element *k* for the *j*-th eigenvector; $B_{k}$ and $D_{k}$ are the strain matrix and elastic matrix of element *k*, respectively; $\sigma_{x}$, $\sigma_{y}$, and $\tau_{xy}$ are the X-direction modal normal stress, Y-direction modal normal stress, and modal shear stress at any point of the element, respectively; $\varepsilon_{x}$, $\varepsilon_{y}$, and $\gamma_{xy}$ are the corresponding modal strains at this point.

The X-direction modal normal strain energy *W_x_* can be calculated by

(4) $$W_{x}=\frac{b}{2}\int_{x}\int_{y}\sigma_{x}\varepsilon_{x}dxdy$$

The Y-direction modal normal strain energy *W_y_* can be calculated by

(5) $$W_{y}=\frac{b}{2}\int_{x}\int_{y}\sigma_{y}\varepsilon_{y}dxdy$$

The modal shear strain energy *W_xy_* can be calculated by

(6) $$W_{xy}=\frac{b}{2}\int_{x}\int_{y}\gamma_{xy}\tau_{xy}dxdy$$

where *b* is the thickness of the element.

The main modal strain energy of the element can be obtained by comparing the values of *W_x_*, *W_y_*, and *W_xy_*. To facilitate the analysis, each modal strain energy corresponds to a color (as shown in Table 1), and this operation is applied to all of the elements of the structure. Thus, the modal strain energy decomposition diagram of the entire structure can be obtained. In addition, a cloud diagram of specific modal strain energy can be drawn according to the magnitude of the energy.

## 3. Experimental Verification

**Figure 1:**
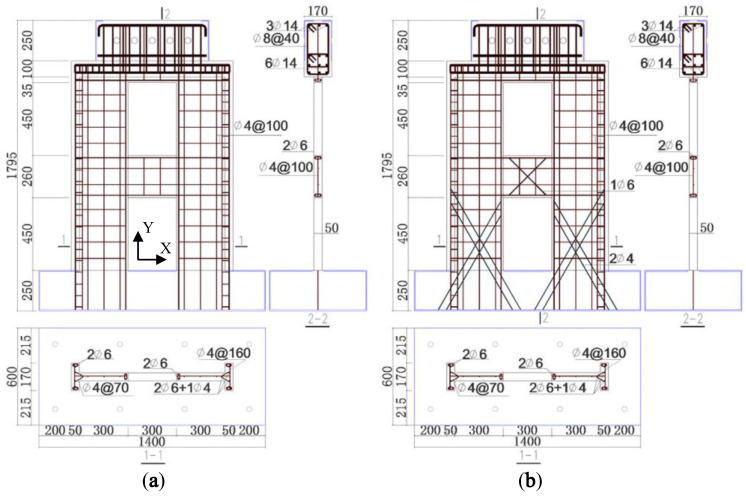
Dimensions and reinforcement of (**a**) A1; (**b**) A2 (unit: mm) [19].

Two-story shear walls A1 and A2 with openings [19] are taken as examples. The dimensional parameters and reinforcement layout of A1 and A2 are shown in Figure 1. The design concrete strength grade of A1 was C30. The elastic modulus of the concrete was 24.43 GPa, and the density was 23.25 kN/m^3^. The walls of A1 had a T-shaped cross-section and were 50 mm thick. The simplified boundary elements around the openings were 2Ø6 longitudinal bars, while the simplified boundary elements for the limbs were columns concealed with triangular stirrups, with 2Ø6 and 1Ø4 longitudinal bars placed [19,20]. The coupling beam and the first-story wall limbs of A2 were additionally provided with 75°-inclined steel bars, and the diameters of the steel bars were Ø6 and Ø4, respectively. The other parameters of A2 were the same as A1. The mechanical properties of the steel bars are shown in Table 2.

**Figure 2:**
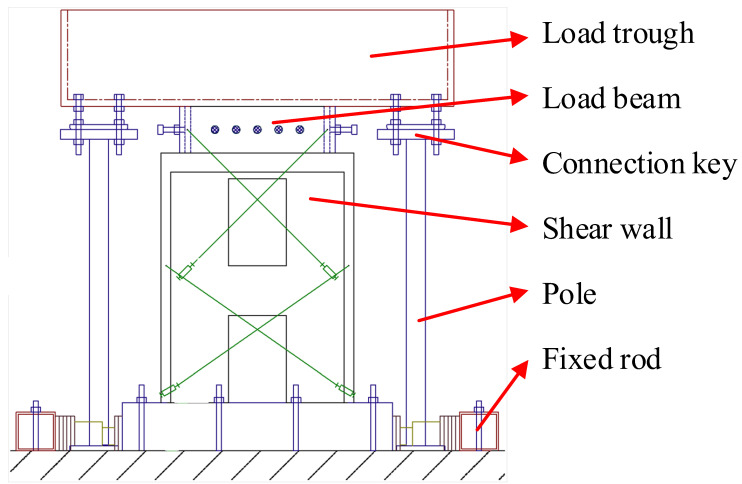
Schematic diagram of test setup [19].

An additional mass of 7.485 tons was applied to the top of A1 and A2 by using a load trough. The specific test setup is shown in Figure 2.

**Figure 3:**
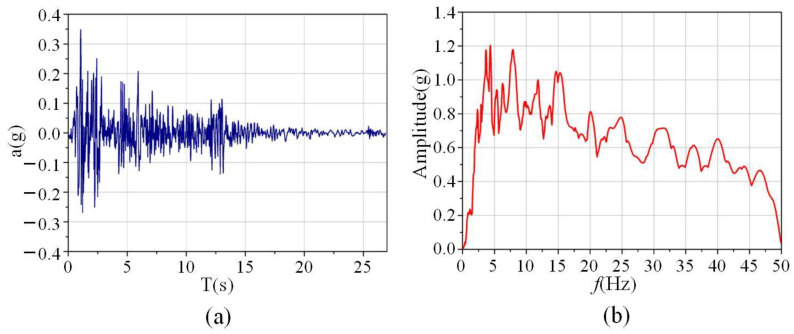
Inputted earthquake: (**a**) time history acceleration; (**b**) response spectra [19].

In the shaking table test in reference [19], the El Centro (1940) N-S wave was used. The scale values of the time interval and duration of the earthquake motion were 0.02 × 0.5 = 0.01 s and 53.74 × 0.5 = 26.87 s, respectively. The time history acceleration and response spectrum of the scaled earthquake motion are shown in Figure 3.

A total of 7 seismic hazard levels were selected and named T-1–T-7. The actual PGA obtained on the shaking table surface during the experiment is shown in Table 3. Using white noise, the initial natural frequencies of shear walls A1 and A2 were 8.25 and 8.30, respectively.

**Figure 4:**
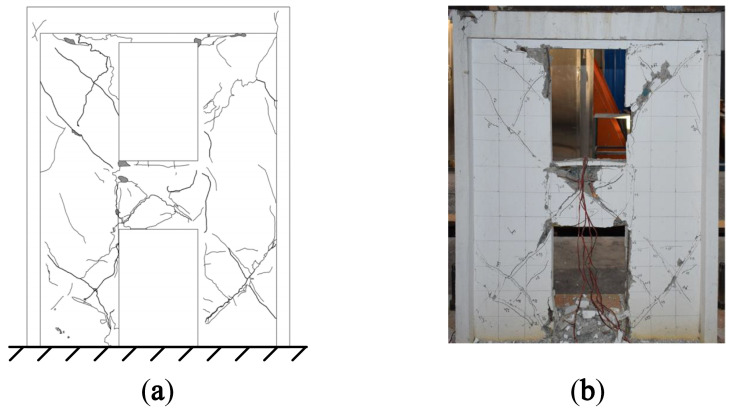
(**a**) crack diagram; (**b**) failure mode of A1 [19].

**Figure 5:**
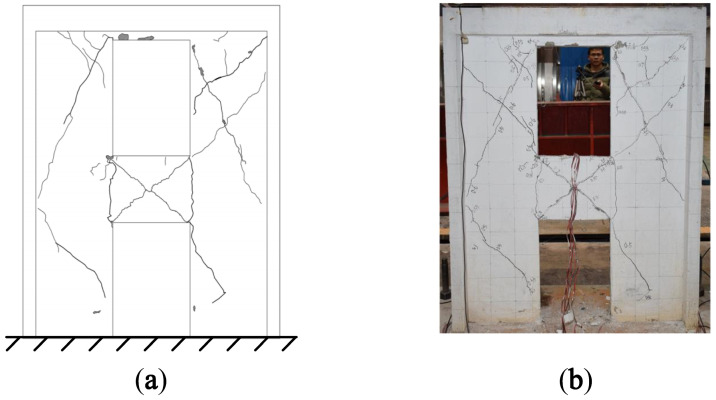
(**a**) crack diagram; (**b**) failure mode of A2 [19].

After the T-6 test, the crack diagrams and failure modes of A1 and A2 are shown in Figure 4 and Figure 5, respectively.

It can be seen from Figure 4 that vertical cracks appeared at the joint areas between the coupling beam and the wall limbs of A1, while the inclined shear cracks were mainly located in the middle of the coupling beam and the wall limbs on both sides of the openings. The middle part of the coupling beam and the corner of the openings were seriously damaged, and there was obvious concrete spalling. Horizontal cracks appeared at the bottom of the wall limb after the T-3 test, and the concrete at the corner of the lower wall limbs was crushed after the T-7 test.

It can be seen from Figure 5 that the locations and types of cracks in A2 were basically the same as those in A1. Due to the constraint effect of the inclined steel bars, the number of inclined cracks in A2 was fewer than that in A1, and the degree of concrete spalling was lesser.

**Figure 6:**
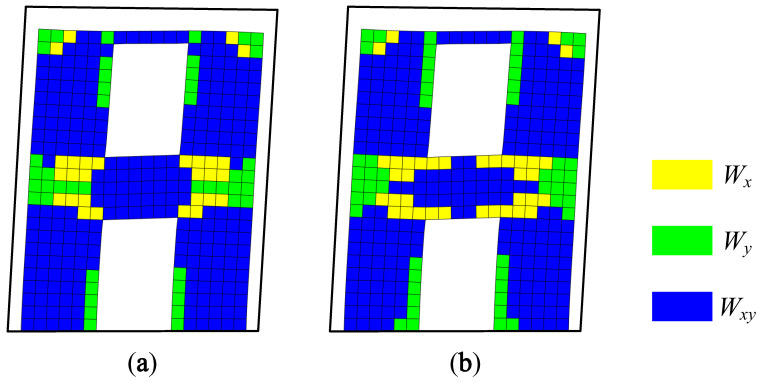
Modal strain energy decomposition diagrams of (**a**) A1; (**b**) A2.

According to the parameters given in reference [19], the finite element models of A1 and A2 were established by ANSYS software. The concrete was established with SOLID65 elements, and the steel bars were established with LINK8 elements. The modeling process used a combination of integral and individual modeling, and the additional mass at the top of the shear wall model was simulated by prestressing. The modal solution was carried out. The first-order natural frequencies of A1 and A2 were 8.54 and 8.66, respectively, which were less than 5% from the measured results. Modal strain energy decomposition was performed on the first-order vibration modes of A1 and A2, and the energy decomposition diagrams are shown in Figure 6.

The modal strain energy decomposition diagram was compared with the crack diagram. The yellow areas (dominated by the X-direction modal normal strain energy) in Figure 6 correspond to the vertical cracks in Figure 4 and Figure 5, while the green areas (dominated by the Y-direction modal normal strain energy) correspond to horizontal cracks, and the blue areas (dominated by the modal shear strain energy) correspond to inclined cracks. The comparison results show that the cracking mode of the structure can be effectively obtained by using the modal strain energy decomposition diagram, which further proves the correctness of the modal strain energy decomposition method.

**Figure 7:**
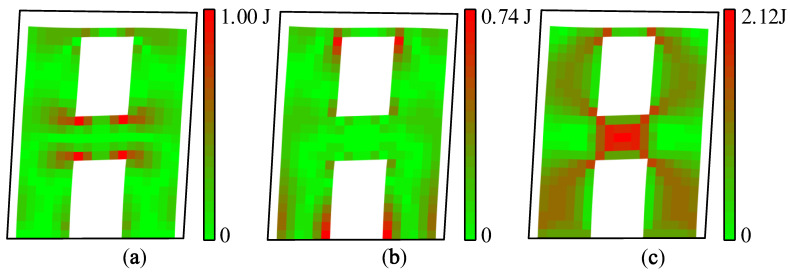
Modal strain energy cloud diagrams of A1: (**a**) X-direction modal normal strain energy, (**b**) Y-direction modal normal strain energy, and (**c**) modal shear strain energy.

**Figure 8:**
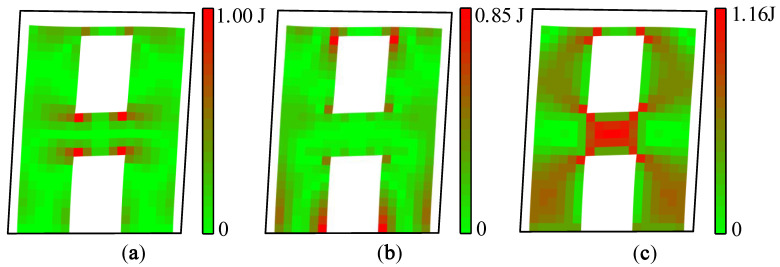
Modal strain energy cloud diagrams of A2: (**a**) X-direction modal normal strain energy, (**b**) Y-direction modal normal strain energy, and (**c**) modal shear strain energy.

Strain, stress, and strain energy can be used to solve the problem of absolute stiffness, such as judging whether a structure will crack under external force and studying the development process of fracturing [21]. The vibration mode vector is the relative value of the nodal displacement. Therefore, the modal strain energy can be used to solve the relative stiffness problems of a structure, such as predicting the location of the first crack and the sequence of cracking so as to realize the purpose of identifying relatively weak areas of a structure. The maximum value of the X-direction modal normal strain energy is regarded as 1.00 J, and each modal strain energy is normalized separately. The modal strain energy cloud diagram of A1 and A2 can be drawn, as shown in Figure 7 and Figure 8.

It can be seen from Figure 7 and Figure 8 that the modal strain energy cloud diagrams of A1 and A2 are similar. The maximum X-direction modal normal strain energy appears at the joint areas between the coupling beam and the wall limbs, and the maximum Y-direction modal normal strain energy appears at the lower wall limbs and the corner of the upper opening. The shear modal strain energy is mainly concentrated in the middle area of the coupling beam, and there is also a relatively obvious energy concentration in the middle of the wall limbs. It is worth noting that the relative value of the modal shear strain energy of A2 was significantly smaller than that of A1.

During the shaking table test, vertical cracks first appeared at the corner of the coupling beam of A1 and gradually increased, and then horizontal cracks appeared at the bottom of the wall limbs. When the PGA was further increased, the coupling beam began to crack obliquely, and the cracks of the wall limbs gradually developed into the inclined shear cracks. Finally, under a seismic excitation with a PGA of 1.219 g, the concrete of the coupling beam of A1 fell off, and the concrete at the corner of the lower wall limbs was crushed.

The cracking sequence of A2 is similar to that of A1. The first crack appeared in the coupling beam, and the wall limbs continued to crack. The damage in the coupling beam was more severe than that in the wall limbs. The difference is that the cracks of A1 were more concentrated in the lower part of the wall limbs, while the cracks of A2 were mainly concentrated in the upper part of the wall limbs, and the number of shear cracks was relatively small in general.

In conclusion, the modal strain energy decomposition diagram and modal strain energy cloud diagram are consistent with the cracking sequence of shear walls A1 and A2, which proves that the modal strain energy decomposition method is an effective way to identify relatively weak areas of a structure. In addition, the process of the modal strain energy decomposition method is in the elastic range and does not involve nonlinear analysis. It is simpler and more convenient than traditional elastic–plastic analytic methods.

## 4. Identification of Relatively Weak Areas of a Structure Based on Different Order Vibration Modes

**Figure 9:**
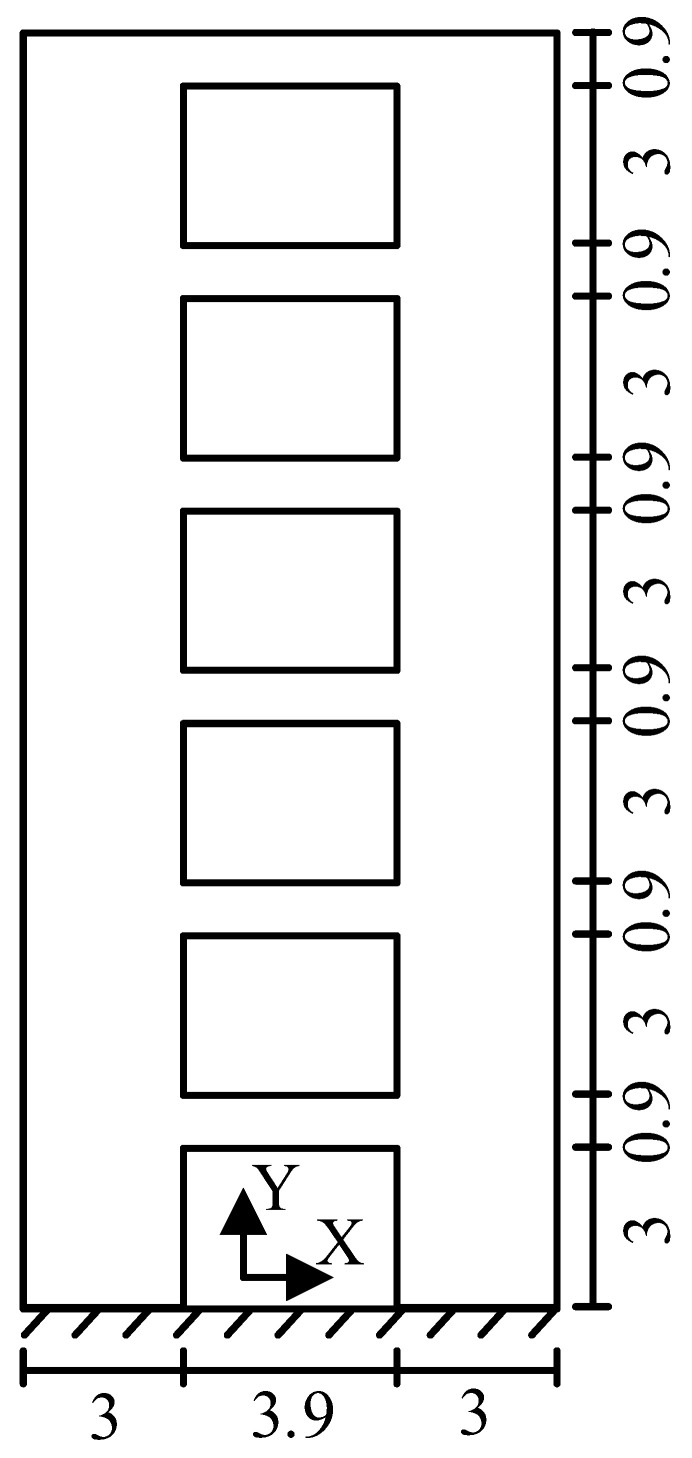
Dimension diagram of shear wall B (unit: m).

Taking 6-story shear wall B with a thickness 0.5 m as an example, its plane size is shown in Figure 9. The elastic modulus of shear wall B is 31.5 GPa, and the density is 2250 kg/m^3^. The finite element model of shear wall B is established with SOLID65 elements, and the number of elements used in the X- and Y-directions are 33 and 78, respectively. According to the principle that “the number of vibration modes can generally be taken as the number required for the participating mass of the vibration modes to reach 90% of the total mass”, the modal strain energy decomposition was carried out for the first two transverse vibration modes of shear wall B.

### 4.1. Identification Result Based on First-Order Vibration Mode

**Figure 10:**
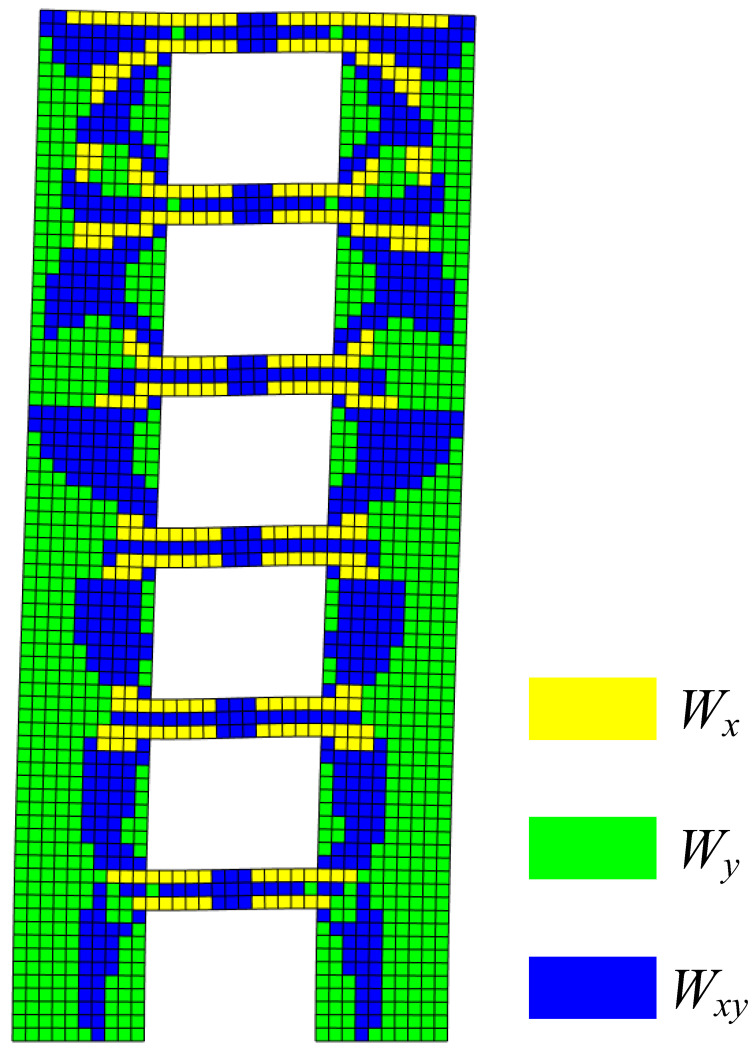
Modal strain energy decomposition diagram of first-order vibration mode.

Modal strain energy decomposition was performed on the first-order vibration mode of shear wall B, and the obtained modal strain energy decomposition diagram is shown in Figure 10.

**Figure 11:**
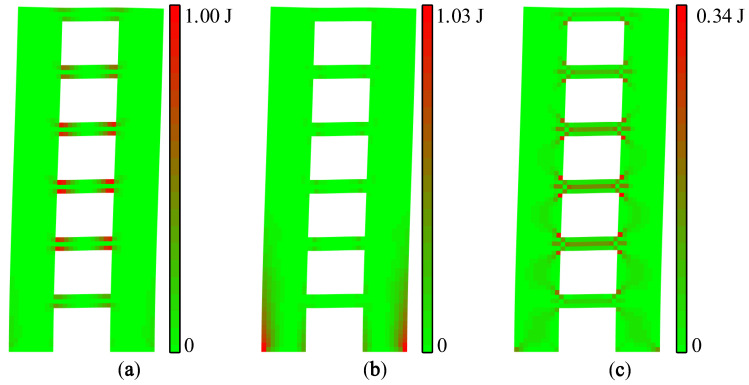
Modal strain energy cloud diagram of first-order vibration mode: (**a**) X-direction modal normal strain energy, (**b**) Y-direction modal normal strain energy, and (**c**) modal shear strain energy.

It can be inferred from Figure 10 that the X-direction modal normal strain energy dominated the joint areas between the coupling beams and the wall limbs, while the Y-direction modal normal strain energy was the main energy of the outer side of the 1st–4th stories. In addition, the inner side of the wall limbs of the 1st–4th stories, the middle areas of the wall limbs of the 5th–6th stories, and the middle areas of the coupling beams were dominated by the modal shear strain energy, and these areas were prone to oblique cracking. In order to further identify the relatively weak areas of shear wall B, the modal strain energy cloud diagram, as shown in Figure 11, was drawn according to the decomposition results.

Figure 11 visualizes the concentration areas of different modal strain energies. It can be seen that the joint areas between the coupling beam and the wall limbs of the 2nd–4th stories are the relatively weak areas of X-direction tension and compression, and vertical cracks will first appear in these areas. The bottom of the wall limbs is a relatively weak area of Y-direction tension and compression; that is, the first horizontal crack will appear at the bottom of shear wall B. There is an obvious modal shear strain energy concentration in the middle of the coupling beams of the 2nd–4th stories, and there is also a certain degree of energy concentration in the middle of the wall limbs of the 1st–3rd stories. It can be inferred that the inclined cracks will first appear at the coupling beams of the 2nd–4th stories, and then gradually develop in the wall limbs.

### 4.2. Identification Results Based on Second-Order Vibration Mode

**Figure 12:**
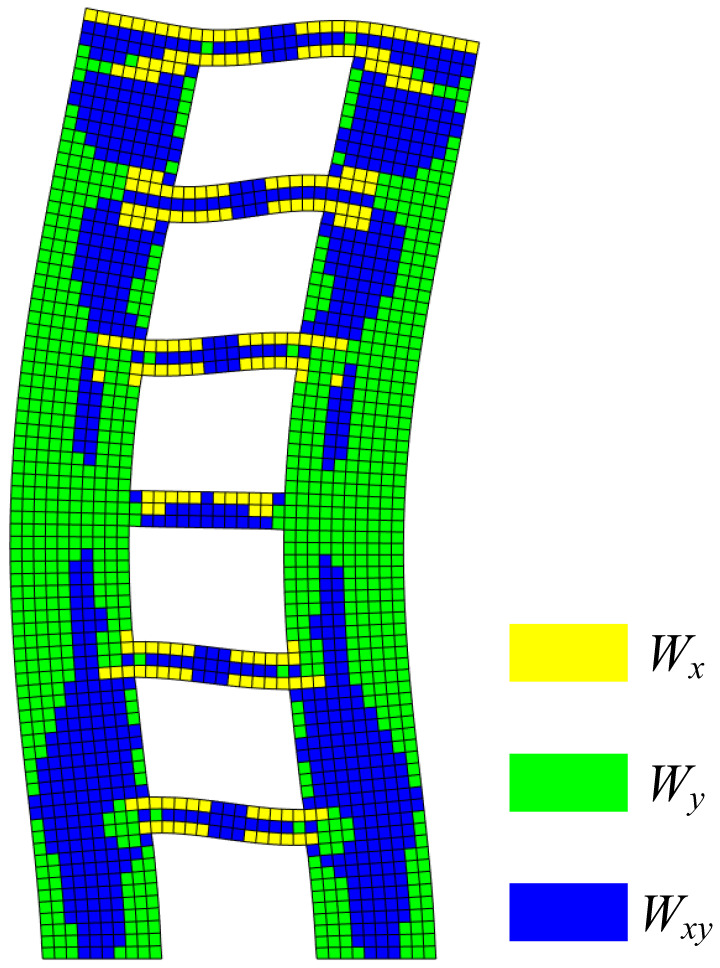
Modal strain energy cloud diagram of second-order vibration mode.

The first-order vibration mode is the most important vibration mode in a structural analysis. However, sometimes the structure is excited to a higher order vibration mode under the action of high-frequency earthquakes. The modal strain energy decomposition of the second-order mode shape of shear wall B was carried out, and the decomposition results are shown in Figure 12.

**Figure 13:**
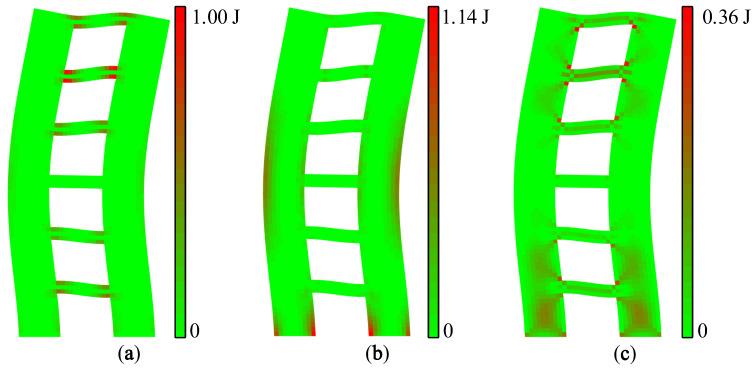
Modal strain energy cloud diagram of second-order vibration mode: (**a**) X-direction modal normal strain energy, (**b**) Y-direction modal normal strain energy, and (**c**) modal shear strain energy.

It can be seen from Figure 12 that the X-direction modal normal strain energy was dominant at both ends of the coupling beam of the B shear wall. The Y-direction modal normal strain energy was the main energy for the 3rd–4th-floor wall. The remaining areas were governed by the modal shear strain energy. According to the decomposition results, the modal strain energy cloud diagrams of shear wall B were drawn, as shown in Figure 13.

For the second-order vibration mode, it can be inferred from Figure 13 that vertical cracks will first appear at the joint areas between the coupling beam and the wall limbs of the 5th story. Horizontal cracks will first appear at the bottom of the wall limbs, and horizontal cracking is also prone to occur on the outer side of the 3rd–4th stories. The modal shear strain energy was mainly concentrated in the coupling beams of the 5th story, and there was also obvious energy concentration in the wall limbs of the 1st–2nd stories, which are prone to oblique shear cracks.

The relatively weak areas of shear wall B under the first two modes are shown in Table 4.

In summary, the modal strain energy decomposition diagram and modal strain energy cloud diagram can intuitively and effectively locate the relatively weak areas of the shear wall structure, predict which parts are prone to cracking, and evaluate the cracking type, which is beneficial to the targeted reinforcement of the structure.

## 5. Conclusions

A modal strain energy decomposition method of planar square elements is proposed and applied to a finite-element analysis of structures. Based on this method, the modal strain energy of a structure can be decomposed, and the quantitative basic modal strain energy information can be obtained. The following conclusions can be drawn:

1. By decomposing the comprehensive modal strain energy of the planar structure into X- and Y-direction modal normal strain energy and modal shear strain energy, the quantitative visual analysis of the vibration mode can be realized.
2. The modal strain energy decomposition diagram can visualize the areas dominated by different modal strain energies, and the cracking mode of a structure can be predicted. The modal strain energy cloud diagram can quantify the concentration degree of different modal strain energies, thereby predicting which areas are prone to cracking and evaluating the cracking type.
3. The modal strain energy decomposition method does involve load condition information and can effectively identify the relatively weak areas of a structure and guide the implementation of targeted reinforcement.

There are still some limitations to this study. In our follow-up work, a strain energy analysis method in the elastic–plastic stage is being developed to realize the real-time evaluation of the deformation state of plastic structures.

## Figures and Tables

**Table 1:** Colors corresponding to the modal strain energy.

X-Direction Modal Normal Strain Energy	Y-Direction Modal Normal Strain Energy	Modal Shear Strain Energy
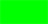	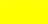	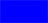

**Table 2:** Mechanical properties of steel bars [19].

Steel Reinforcement Diameter (mm)	Yield Strength (MPa)	Ultimate Strength (MPa)	Elastic Modulus (GPa)
Ø4	730.3	903.0	190.6
Ø6	394.5	578.3	220.7

**Table 3:** Test procedure [19].

Shear Walls	PGA (g)						
	T-1	T-2	T-3	T-4	T-5	T-6	T-7
A1	0.189	0.388	0.524	0.615	0.815	1.155	1.219
A2	0.191	0.330	0.492	0.650	0.782	0.875	1.126

**Table 4:** Relatively weak areas of shear wall B under the first- and second-order vibration modes.

Order	Tension and Compression		Shear
	X-Direction	Y-Direction	
1st	joint areas between coupling beam and wall limbs of the 2nd–4th stories	bottom of the wall limbs	coupling beams of the 2nd–4th stories, and the wall limbs of the 1st–3rd stories
2nd	joint areas between coupling beam and wall limbs of the 5th story	bottom of the wall limbs, and outer side of the 3rd–4th stories	coupling beams of the 5th story, and the wall limbs of the 1st–2nd stories

## Data Availability

Some or all data that support the findings of this study are available from the corresponding author upon reasonable request.
